# SIRT1 and SIRT3 Deacetylate Homologous Substrates: AceCS1,2 and HMGCS1,2

**DOI:** 10.18632/aging.100339

**Published:** 2011-06-19

**Authors:** Matthew D. Hirschey, Tadahiro Shimazu, John A. Capra, Katherine S. Pollard, Eric Verdin

**Affiliations:** ^1^ Gladstone Institute of Virology and Immunology, University of California, San Francisco, CA 94158; ^2^ Gladstone Institute of Cardiovascular Disease, University of California, San Francisco, CA 94158; ^3^ Division of Biostatistics & Institute for Human Genetics, University of California, San Francisco, CA 94107; ^4^ Department of Medicine, University of California, San Francisco, CA 94143

**Keywords:** sirtuins, evolution, deacetylases, aging

## Abstract

SIRT1 and SIRT3 are NAD+-dependent protein deacetylases that are evolutionarily conserved across mammals. These proteins are located in the cytoplasm/nucleus and mitochondria, respectively. Previous reports demonstrated that human SIRT1 deacetylates Acetyl-CoA Synthase 1 (AceCS1) in the cytoplasm, whereas SIRT3 deacetylates the homologous Acetyl-CoA Synthase 2 (AceCS2) in the mitochondria. We recently showed that 3-hydroxy-3-methylglutaryl CoA synthase 2 (HMGCS2) is deacetylated by SIRT3 in mitochondria, and we demonstrate here that SIRT1 deacetylates the homologous 3-hydroxy-3-methylglutaryl CoA synthase 1 (HMGCS1) in the cytoplasm. This novel pattern of substrate homology between cytoplasmic SIRT1 and mitochondrial SIRT3 suggests that considering evolutionary relationships between the sirtuins and their substrates may help to identify and understand the functions and interactions of this gene family. In this perspective, we take a first step by characterizing the evolutionary history of the sirtuins and these substrate families.

## INTRODUCTION

**Figure 1:**
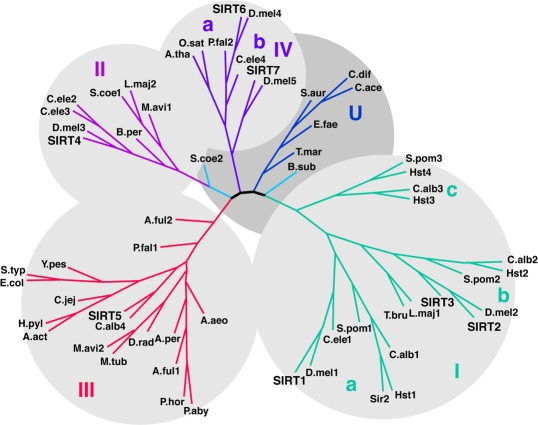
Phylogenetic analysis of the sirtuins. An unrooted tree diagram of the phylogenetic analysis of sirtuin domain sequences, divided into Class I, II, III, IV and U groups. Classes I and IV are further divided into subclasses indicated by lowercase letters. Organism abbreviations: A. act, *Actinobacillus actinomycetemcomitans*; A. aeo, *Aquifex aeolicus*; A. ful, *Archaeoglobus fulgidus*; A. per, *Aeropyrum pernix*; A. tha, *Arabidopsis thaliana*; B. per, *Bordetella pertussis*; B. sub, *Bacillus subtilis*; C. ace, *Clostridium acetabutylicum*; C. alb, *Candida albicans*; C. dif, *Clostridium difficile*; C. ele, *Caenorhabditis elegans*; C. jej, *Campylobacter jejuni*; D. mel, *Drosophila melanogaster*; D. rad, *Deinococcus radiodurans*; E. col, *Escherichia coli*; E. fae, *Enterococcus faecalis*; H. sap, *Homo sapiens*; H. pyl, *Helicobacter pylori*; L. maj, *Leishmania major*; M. avi, *Mycobacterium avium*; M. tub, *Mycobacterium tuberculosis*; O. sat, *Oryza sativa*; P. aby, *Pyrococcus abyssi*; P. fal, *Plasmodium falciparum*; P. hor, *Pyrococcus horikoshii*; S. aur, *Staphylococcus aureus*; S. coe, *Streptomyces coelicolor*; S. pom, *Schizosaccharomyces pombe*, S. typ, *Salmonella typhimurium*; S. cer, *Saccharomyces cerevisiae*; T. bru, *Trypanosoma brucei*; T. mar, *Thermotoga maritima*; Y. pes, *Yersinia pestis*. Adapted from [7].

Sirtuins are a family of NAD^+^-dependent protein deacetylases/ADP ribosyltransferases that target a wide range of cellular proteins involved in aging [1-3], DNA repair [4], and metabolic regulation [5]. The sirtuins are present across the tree of life. The number of sirtuins in different species' genomes ranges from one in *E. coli* (CobB) to seven in mammals (SIRT1-7). A yeast sirtuin family protein Sir2p functions as an histone deacetylase, and regulates replicative senescence and life span [3]. Among the seven mammalian sirtuins, SIRT1, the closest homologue of yeast Sir2p, is found in the cytoplasm and nucleus and plays diverse physiological roles in cellular signaling, transcriptional regulation (reviewed in [6]). Its orthologs, Sir2.1 and dSIR2, play similar roles in worms [2] and flies [1], respectively. Clustering of the sirtuins based on sequence similarity produces four general classes of sirtuins (Figure 1) [7]. Of all sirtuins, Class I sirtuins (SIRT1, SIRT2, SIRT3 in mammals) exhibit the most robust deacetylase activity on a variety of natural and synthetic acetylated substrates. Class II sirtuins (SIRT4) have no detectable deacetylase activity and instead show weak ADP-ribosyltransferase activity [8, 9]; class III sirtuins (SIRT5) have only weak deacetylase activity on the histone substrate [10, 11]; class IV sirtuins have ADP ribosyltransferase and deacetylase activity (SIRT6) or unknown activity (SIRT7) [12, 13]. An additional class of sirtuins referred to as Class U is intermediate between Class I and IV and has only been observed in bacteria. Of the human mitochondrial sirtuins, SIRT3 has the most similar sequence to SIRT1, so we investigated the possibility that SIRT3 regulates similar functions in the mitochondria as SIRT1 regulates in the cytoplasm.

**Figure 2:**
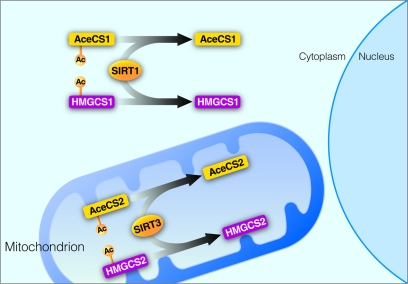
Regulation of homologous enzymes by sirtuins SIRT1 and SIRT3. SIRT1 deacetylates acetyl-CoA synthetase 1 (AceCS1) and 3-hydroxy-3-methylglutaryl CoA synthase 1 (HMGCS1) in the cytoplasm. SIRT3 deacetylates acetyl-CoA synthetase 2 (AceCS2) and 3-hydroxy-3-methylglutaryl CoA synthase 2 (HMGCS2) in the mitochondrial matrix.

The first identified substrate of SIRT3 is acetyl-CoA synthetase 2 (AceCS2) [14, 15]. In mammalian cells, two acetyl-CoA synthase enzymes are known: AceCS1 [16] and AceCS2 [17]. AceCS2 is located in mitochondria and catalyzes the activation of acetate, ATP and CoA into acetyl-CoA for TCA cycle oxidation in extra-hepatic tissues [18]. AceCS1 is found in the cytoplasm and there catalyzes the conversion of acetate, ATP and CoA into acetyl-CoA for fatty acid and lipid biosynthesis [16, 19]. Both AceCS1 and AceCS2 are acetylated on the same site, however their deacetylation is mediated by different sirtuins: cytoplasmic SIRT1 for AceCS1 and mitochondrial SIRT3 for AceCS2 [14, 15]. Thus, not only do the mitochondrial and cytoplasmic AceCS perform similar molecular functions, their regulation via acetylation is conserved and mediated by two sirtuins with distinct subcellular localization (Figure 2).

### Both Mitochondrial and Cytoplasmic Hydroxy-3-methylglutaryl CoA synthases (HMGCS) are Regulated by Sirtuins

The enzyme 3-hydroxy-3-methylglutaryl CoA synthase 2 (HMGCS2) catalyzes the conversion of acetyl-CoA and acetoacetyl-CoA into 3-hydroxymethylglutarylCoA and represent the rate-limiting step in ketone body synthesis [20]. HMGCS2 is acetylated at 9 lysine residues and the acetylation of 3 of these sites (310, 447, and 473) increases in the absence of SIRT3, which reduces its enzymatic activity. During fasting, SIRT3 expression increases, leading to the deacetyation of HMGCS2 and to an increase in its enzymatic activity. Molecular dynamics simulations of wild-type and hyperacetylated HMGCS2 show that *in silico* deacetylation of these three lysines cause conformational changes of HMGCS2 near the active site and positions two important catalytic residues closer to their substrate acetyl-CoA.

Interestingly, there is also a cytoplasmic homolog of HMGCS2 called HMGCS1, a critical enzyme in cholesterol synthesis (for a review, see [21]). Because SIRT1 and SIRT3 were previously shown to deacetylate homologous substrates in the cytoplasm and mitochondria, respectively, we tested the possibility that SIRT1 might deacetylate HMGCS1.

First, we aligned the protein sequences of HMGCS1 and HMGCS2, for both mouse and humans. We found 83% similarity between human HMGCS1 and HMGCS2, and 68% identical residues (Figure S1). Additionally, we found 84% similarity between mouse HMGCS1 and HMGCS2, and 66% identical residues (Figure S1). Several lysine residues were conserved between HGMCS1 and HMGCS2, including one conserved lysine (K310) targeted by SIRT3 on HMGCS2. Thus, HMGCS1 was a strong candidate for regulation by acetylation.

**Figure 3:**
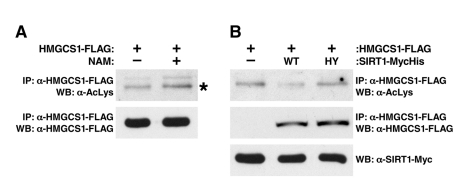
SIRT1 Regulates the Acetylation Level of HMGCS1. (**A**) Recombinant HMGCS1 was expressed in HEK293 cells in the absence (control) or presence of nicotinamide (NAM, 50 mM), purified and measured for acetylation with an anti-acetyllysine antibody. (**B**) Expression vectors for wt SIRT1 or catalytically inactive SIRT1-H363Y were co-transfected into HEK293 cells with expression vectors for FLAG-tagged HMGCS1, and the levels of HMGCS1 acetylation were assessed.

To test if HMGCS1 is regulated by a sirtuin, we measured its acetylation level in cells treated with the sirtuin inhibitor nicotinamide (NAM). An FLAG-tagged HMGCS1 protein was expressed in human HEK293 cells in the absence (control) or presence of NAM, immunoprecipitated and assessed for lysine acetylation. HMGCS1 acetylation increased upon NAM treatment in mammalian cells, suggesting a cytoplasmic sirtuin regulates the acetylation status of HMGCS1 (Figure 3A).

To test the hypothesis that SIRT1 directly deacetylates HMGCS1, expression vectors encoding FLAG-tagged HMGCS1 were co-transfected with expression vectors for SIRT1 or catalytically inactive SIRT1-H363Y mutant (HA-tagged) into HEK293 cells. HMGCS1 acetylation levels were assessed after immunoprecipitation (anti-FLAG) and western blotting with an anti-acetyllysine antiserum. We found SIRT1, but not catalytically inactive SIRT1-H363Y, deacetylates HMGCS1 (Figure 3B). This is the second example of SIRT1 deacetylating one substrate (HMGCS1) in the cytoplasm, while SIRT3 deacetylates its homolog (HGMCS2) in the mitochondria. This observation suggests the possibility of a more general pattern of evolutionary relatedness between the substrates of SIRT1 and SIRT3.

### Evolutionary history of the sirtuins and their substrates

Families of multiple homologous genes within a species, such as the sirtuins, HMGCS1/HMGCS2, and AceCS1/AceCS2, are created by gene duplications [22]. By analyzing the sequences of the family members present in the genomes of species across the tree of life, the evolutionary history of gene origin, duplication, and loss for a family can be inferred [23]. Understanding when in evolutionary time genes first appeared and subsequently duplicated provides useful context for understanding their function, interactions, and evolution.

**Figure 4:**
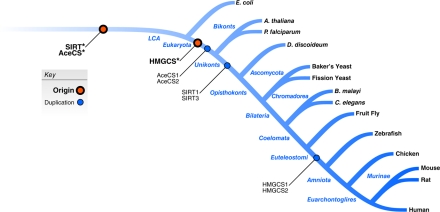
Evolutionary analysis of human sirtuins and their known homologous substrates. Gene family origin and duplication events for SIRT1/SIRT3 and two known homologous substrate families are plotted on the phylogenetic tree. Family origins are indicated by red circles, and duplication events are shown as blue circles.

To characterize the evolutionary relationships between SIRT1 and SIRT3 and their substrates, we performed a phylogenetic analysis of these families. In these analyses, evolutionary events are assigned to branches on the tree of life (Figure 4), and these branches are referred to by the name of their child node in the tree. For example, if a gene duplicated after the last common ancestor (LCA) of humans and yeast, but before the LCA of human and worms, it would be assigned to the “Bilateria” branch. We found that the sirtuins and acetyl-CoA synthase enzymes (AceCS*) appeared very early in the history of life; precursors of these families were present in the LCA of all cellular organisms (Figure 4). The ancient origin of the acetylation machinery and its presence in the vast majority of modern-day organisms argues that enzymatic regulation by acetylation and deacetylation is an important, conserved regulatory mechanism. Indeed, recent evidence points to the presence of numerous acetylated proteins in *E. coli* [24] and in human liver cells [25], and a role for acetylation in metabolic regulation in these organisms.

The conserved deacetylation of these two pairs of homologous substrates by SIRT3 in the mitochondrion and SIRT1 in the cytoplasm suggests that these gene families may have evolved in a coordinated fashion. To investigate this hypothesis further, we reconciled the species tree with gene trees for the sirtuins, AceCS1/AceCS2, and HMGCS1/HMGCS2. We then estimated the timing of duplication and loss events within these families. As expected, the seven human members of the sirtuin family experienced many duplications and losses over the course of their evolution. The duplication that created the ancestors of SIRT1 and SIRT3 is predicted to have occurred early in the evolution of the first eukaryotes (Figure 4). Consistent with the possible coevolution of these families, the AceCS1/AceCS2 duplication is also quite ancient and was present in the LCA of all eukaryotes. In contrast, the HMGCS1/HMGCS2 duplication event occurred much later, around the origin of vertebrates (Figure 4). From this observation, we predict the full regulatory program of the current sirtuins has expanded since their ancestral counterparts and that current patterns of enzymatic regulation by acetylation evolved over a long period of time.

### Conclusion

We performed an evolutionary analysis of the sirtuins and recently identified substrates. We observed a homologous relationship between substrates of cytoplasmic SIRT1 and mitochondrial SIRT3. While all homologous gene pairs that localize to the cytoplasm and mitochondria are unlikely to be substrates of SIRT1 and SIRT3, we speculate that more homologous substrates will be found as more substrates of SIRT1 and/or SIRT3 are identified. In fact, when new substrates of SIRT1 or SIRT3 are identified, this relationship can be used to identify potential substrates of the homologous sirtuin. These evolutionary relationships suggest that phylogenetic analyses can be combined with experimental data to better understand the functions of this important gene family.

## EXPERIMENTAL PROCEDURES

### Cell Culture and Plasmid Construction

HEK293 cells were cultured in DMEM supplemented with 10% FCS. All expression constructs were generated by standard PCR-based cloning strategies, and all expression constructs were verified by DNA sequencing. The human HMGCS1 coding sequence was PCR-amplified from human full-length Mammalian Gene Collection cDNA (GenBank accession no. NM_002130; obtained through Open Biosystems, Huntsville, AL) and cloned into the pcDNA3.1+ derived vectors pcDNA-Flag to yield HMGCS1 with a C-terminal Flag (Invitrogen, Carlsbad, CA). Human SIRT1 was also cloned into pcDNA-Flag or pcDNA-HA.

### Immunoprecipitation & Immunoblotting

Cells were lysed on ice in NP1 buffer (PBS with 0.5% Nonidet P-40 and 0.2 mM PMSF) with protease inhibitor cocktail (Roche). Flag-tagged proteins were immunoprecipitated and washed in NP1 buffer four times. Antibodies used were anti-Flag M2 or rabbit polyclonal anti-Flag (Sigma-Aldrich, St. Louis, MO), acetylated-lysine polyclonal antibody (Cell Signaling Technology, Danvers, MA). Immunoblots were developed with enhanced chemiluminescence (Amersham Pharmacia Biosciences, Piscataway, NJ) or West SuperSignal reagent (Pierce, Rockford, IL).

### Bioinformatic and Phylogenetic Analyses

We predicted the ages of genes following a strategy similar to the phylostratigraphic method of [26]. We downloaded homologous protein families predicted using the Naïve Ensemble method of the Princeton Protein Orthology Database Version 4 [27], which combines the predictions of OrthoMCL [28] and InParanoid [29] across the genomes listed in Figure 4. We analyzed the phylogenetic distribution of orthologs for each human gene. Each gene was assigned the node in the species tree that was the lowest common ancestor of all of its orthologs as its age. Duplication histories were inferred using Notung [30] to reconcile gene and species trees.

## SUPPLEMENTAL MATERIAL

Figure S1 Multiple sequence alignment of HMGCS1 and HMGCS2. Mouse and human protein sequences of HMGCS1 and HMGCS2 (NP_002121, NP_005509, NP_666054, NP_032282) were aligned and evaluated for conservation and identity using BlastP. Acetylated lysine residues identified on HMGCS2 are indicated by triangles.
