# Supplementary figures and images for: SIRT1 and SIRT3 Deacetylate Homologous Substrates: AceCS1,2 and HMGCS1,2
Source: Aging (Albany NY). 2011 Jun 19;3(6):635–42. doi: 10.18632/aging.100339 (PMC3164371; doi:10.18632/aging.100339)

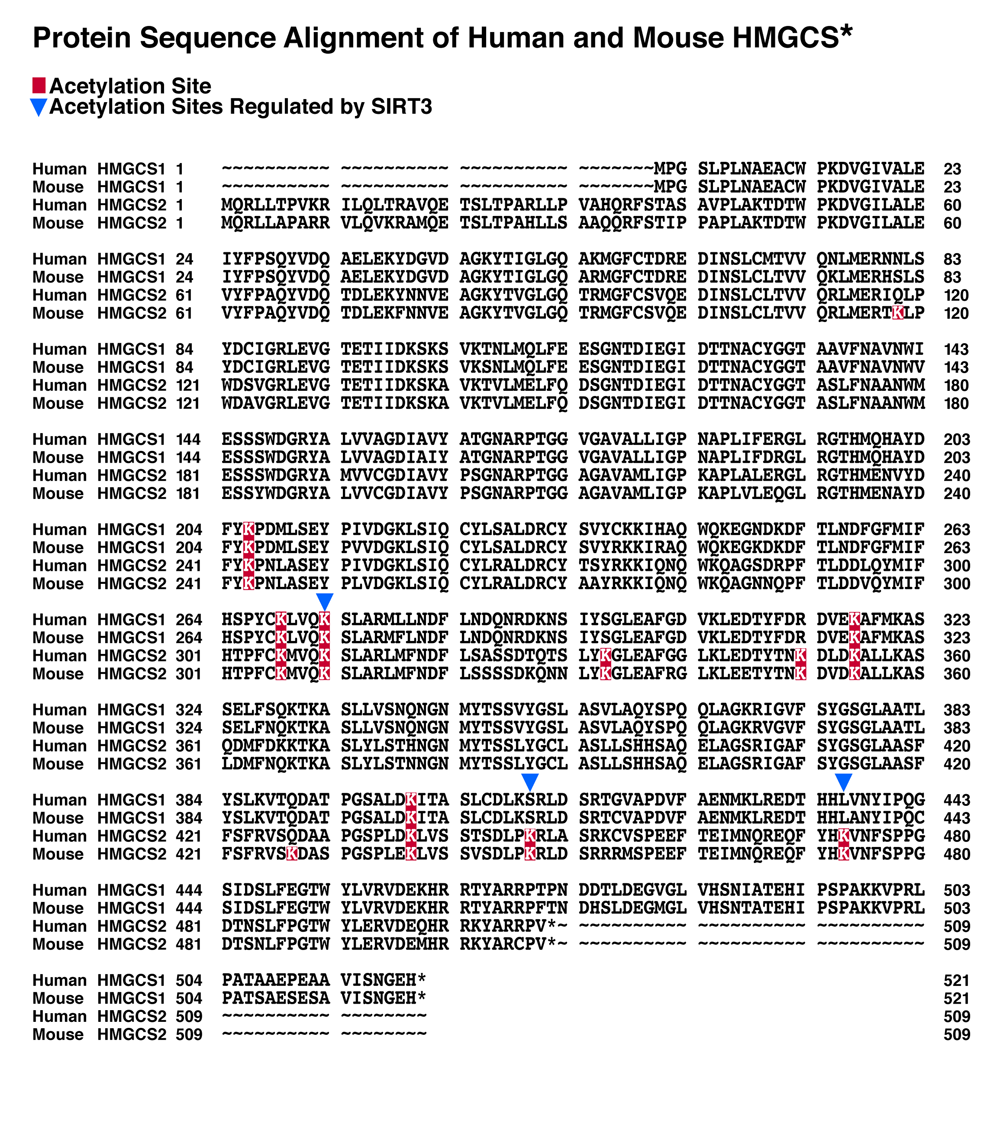

Supplement: Figure S1 — Mouse and human protein sequences of HMGCS1 and HMGCS2 (NP_002121, NP_005509, NP_666054, NP_032282) were aligned and evaluated for conservation and identity using BlastP. Acetylated lysine residues identified on HMGCS2 are indicated by triangles. [file aging-03-635-s001.tif]
